# Supplementary material for: Fasciculation potentials are related to the prognosis of amyotrophic lateral sclerosis
Source: PLoS One. 2024 Nov 8;19(11):e0313307. doi: 10.1371/journal.pone.0313307 (PMC11548741; doi:10.1371/journal.pone.0313307)
Supplement: S1 Fig — (DOCX) [file pone.0313307.s001.docx]

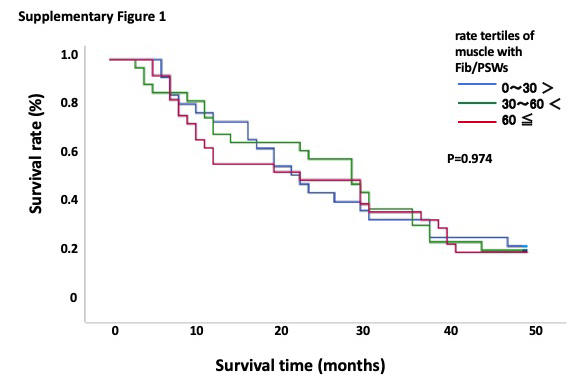


Supplementary Figure 1. Comparison of the survival curves after disease onset stratified by rate of muscle with fibrillation potentials and positive sharp waves (Fib/PSWs) in ALS patients by using Kaplan–Meier method.
