# Supplementary material for: Fasciculation potentials are related to the prognosis of amyotrophic lateral sclerosis
Source: PLoS One. 2024 Nov 8;19(11):e0313307. doi: 10.1371/journal.pone.0313307 (PMC11548741; doi:10.1371/journal.pone.0313307)
Supplement: S2 Fig — (DOCX) [file pone.0313307.s002.docx]

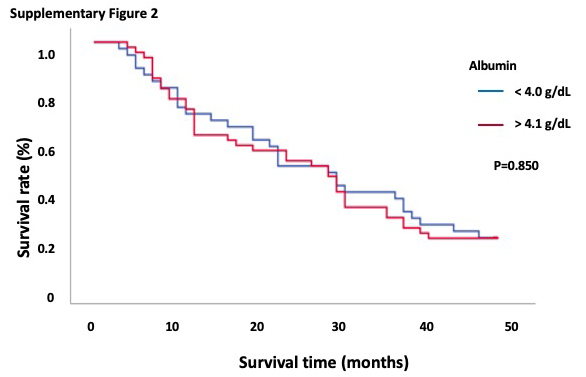


Supplementary Figure 2. The survival curves for ALS patients with albumin < 4 g/dL vs. albumin > 4.1 g/dL using Kaplan–Meier method.
