# Supplementary material for: Fasciculation potentials are related to the prognosis of amyotrophic lateral sclerosis
Source: PLoS One. 2024 Nov 8;19(11):e0313307. doi: 10.1371/journal.pone.0313307 (PMC11548741; doi:10.1371/journal.pone.0313307)
Supplement: S3 Fig — The survival curves for male ALS patients with total cholesterol < 189 mg/dL vs. total cholesterol > 190 mg/dL using Kaplan–Meier method (A). The survival curves for female ALS patients with total cholesterol < 199 mg/dL vs. total cholesterol > 200 mg/dL using Kaplan–Meier method (B). (DOCX) [file pone.0313307.s003.docx]

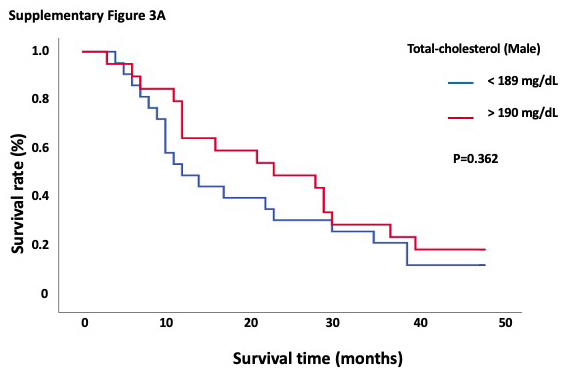


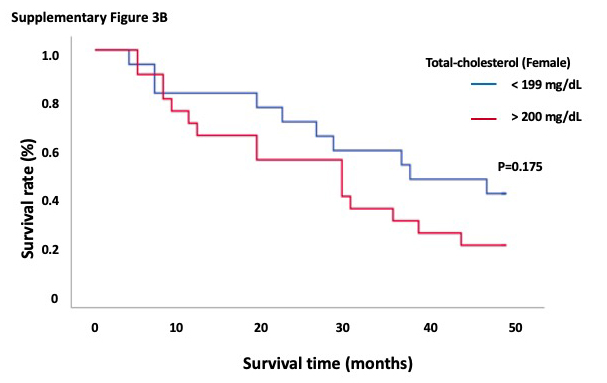


Supplementary Figure 3. The survival curves for male ALS patients with total cholesterol < 189 mg/dL vs. total cholesterol > 190 mg/dL using Kaplan–Meier method (A). The survival curves for female ALS patients with total cholesterol < 199 mg/dL vs. total cholesterol > 200 mg/dL using Kaplan–Meier method (B).
