# Supplementary material for: Fasciculation potentials are related to the prognosis of amyotrophic lateral sclerosis
Source: PLoS One. 2024 Nov 8;19(11):e0313307. doi: 10.1371/journal.pone.0313307 (PMC11548741; doi:10.1371/journal.pone.0313307)
Supplement: S4 Fig — The survival curves for male ALS patients with creatine kinase < 180 U/L vs. creatine kinase > 181 U/L using Kaplan–Meier method (A). The survival curves for female ALS patients with creatine kinase < 80 U/L vs. creatine kinase > 81 U/L using Kaplan–Meier method (B). (DOCX) [file pone.0313307.s004.docx]

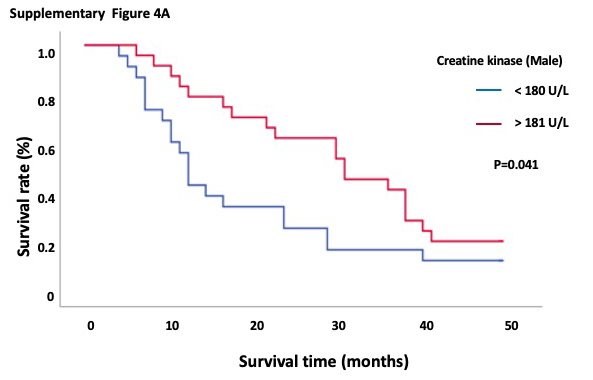


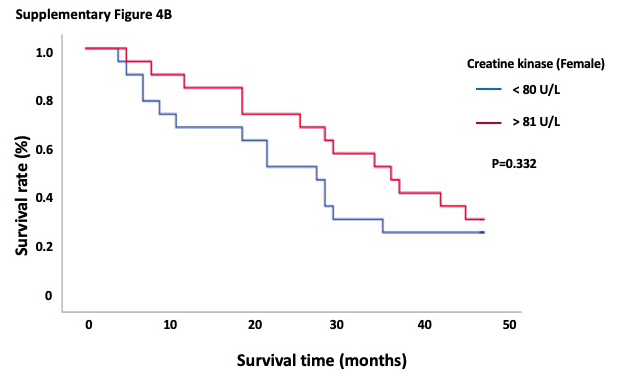


Supplementary Figure 4. The survival curves for male ALS patients with creatine kinase < 180 U/L vs. creatine kinase > 181 U/L using Kaplan–Meier method (A). The survival curves for female ALS patients with creatine kinase < 80 U/L vs. creatine kinase > 81 U/L using Kaplan–Meier method (B).
