# Supplementary material for: Fasciculation potentials are related to the prognosis of amyotrophic lateral sclerosis
Source: PLoS One. 2024 Nov 8;19(11):e0313307. doi: 10.1371/journal.pone.0313307 (PMC11548741; doi:10.1371/journal.pone.0313307)
Supplement: S5 Fig — (DOCX) [file pone.0313307.s005.docx]

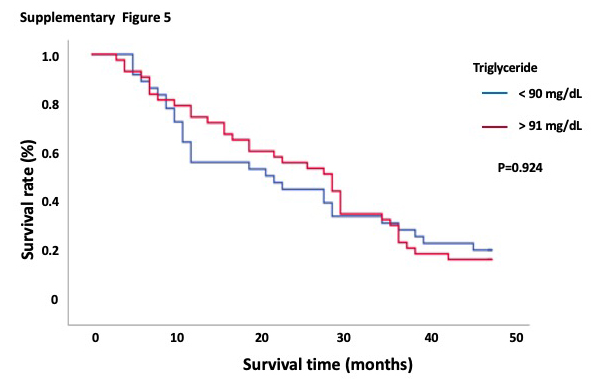


Supplementary Figure 5. The survival curves for ALS patients with triglyceride < 90 mg/dL vs. triglyceride > 91 mg/dL using Kaplan–Meier method.
