# Supplementary material for: Fasciculation potentials are related to the prognosis of amyotrophic lateral sclerosis
Source: PLoS One. 2024 Nov 8;19(11):e0313307. doi: 10.1371/journal.pone.0313307 (PMC11548741; doi:10.1371/journal.pone.0313307)
Supplement: S6 Fig — The survival curves for male ALS patients with creatinine < 0.6 mg/dL vs. creatinine > 0.61 mg/dL by using Kaplan–Meier method (A). The survival curves for female ALS patients with creatinine < 0.5 mg/dL vs. creatinine > 0.51 mg/dL by using Kaplan–Meier method (B). (DOCX) [file pone.0313307.s006.docx]

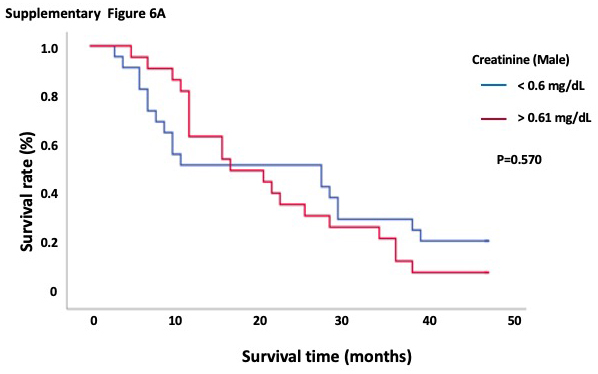


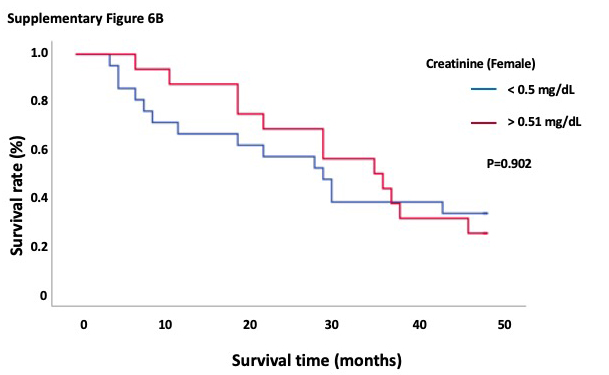


Supplementary Figure 6. The survival curves for male ALS patients with creatinine < 0.6 mg/dL vs. creatinine > 0.61 mg/dL by using Kaplan–Meier method (A). The survival curves for female ALS patients with creatinine < 0.5 mg/dL vs. creatinine > 0.51 mg/dL by using Kaplan–Meier method (B).
