# Supplementary material for: Fasciculation potentials are related to the prognosis of amyotrophic lateral sclerosis
Source: PLoS One. 2024 Nov 8;19(11):e0313307. doi: 10.1371/journal.pone.0313307 (PMC11548741; doi:10.1371/journal.pone.0313307)
Supplement: S7 Fig — (DOCX) [file pone.0313307.s007.docx]

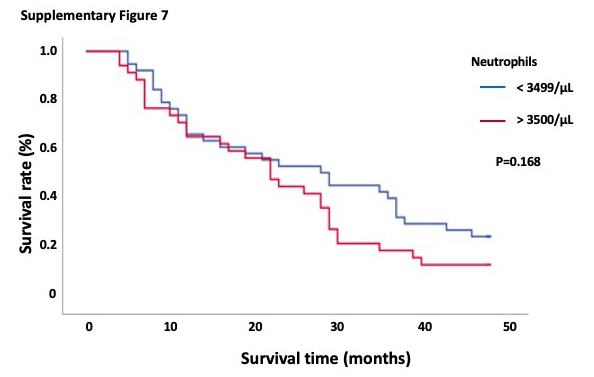


Supplementary Figure 7. The survival curves for ALS patients with neutrophils < 3499/μL vs. neutrophils > 3500/μL using Kaplan–Meier method.
