# Supplementary material for: Fasciculation potentials are related to the prognosis of amyotrophic lateral sclerosis
Source: PLoS One. 2024 Nov 8;19(11):e0313307. doi: 10.1371/journal.pone.0313307 (PMC11548741; doi:10.1371/journal.pone.0313307)
Supplement: Attachment — Submitted filename: Reviewer-Comments.pdf [file pone.0313307.s008.pdf]

PONE-D-24-18589: Fasciculation potentials are related to the prognosis of amyotrophic lateral sclerosis

The manuscript presents a study investigating the relationship between the proportion of muscles with fasciculation potentials (FPs), various biochemical markers, and the prognosis of ALS. The findings are significant and well-supported by the data, demonstrating clear associations between the percentage of muscles with FPs, uric acid (UA) levels, and ALS progression.With the suggested major revisions and clarifications, the manuscript will be well-positioned to make a substantial contribution to the field of ALS research.

Below are my comments:

Major comments

1. The manuscript is missing one of the most important progressions measuring tool, **ALS Functional Rating Scale-Revised (ALSFRS-R)**. This tool is widely used clinical tool that assesses functional abilities across four domains: bulbar function, fine motor function, gross motor function, and respiratory function. The ALS FR should be provided for all patients and the disease progression and the findings in this manuscript should be clearly correlated. Include data on functional outcomes using ALSFRS-R scores to correlate the findings with patient quality of life and functional decline. This will help validate the clinical significance of FPs and UA levels.

2. Assess the firing frequency and other characteristics of FPs. This could differentiate between benign fasciculations and those indicative of ALS progression. Such detailed analysis can add depth to the understanding of FPs in ALS.

3. Is the genetic data available for these patients? Most importantly the commonly associated mutations with ALS (e.g., SOD1, C9orf72) to see if there is any correlation with FPs and UA levels. This could provide insights into the underlying mechanisms.

4. The control data is missing. Include a healthy control group for comparison to clearly delineate the differences in FPs and UA levels between ALS patients and healthy individuals. This will strengthen the argument for the specificity of these markers in ALS. Comparisons should be made between healthy controls and patients for making any associations between findings.

5. The discussion needs more details of the potential mechanisms underlying the associations found.

Minor comments

1. Consider providing a brief overview of ALS, its pathophysiology, and the significance of identifying prognostic markers.

2. The rationale for focusing on FPs and UA levels is well-articulated, but it might benefit from a brief explanation of how these markers have been studied in previous studies.

3. Clearly classify the patients are possible, probable and definite ALS.

4. Mention the specific criteria used for defining FPs in more detail, including any relevant thresholds or cut-off values.

5. Address the study's limitations more comprehensively, including potential biases and generalizability issues.

6. Suggest directions for future research, particularly regarding the clinical application of your findings.
