# Supplementary material for: Fasciculation potentials are related to the prognosis of amyotrophic lateral sclerosis
Source: PLoS One. 2024 Nov 8;19(11):e0313307. doi: 10.1371/journal.pone.0313307 (PMC11548741; doi:10.1371/journal.pone.0313307)
Supplement: Attachment — Submitted filename: Response_to_Reviewer.docx [file pone.0313307.s010.docx]

Reviewer #1: The authors have done a commendable job with the revisions, but I would like to point out a minor issue in the following sentence: 'Pathological sensory disturbance detected through skin biopsies has also been reported for ALS, with observations including axonal swellings as the foremost morphological changes and small fiber regeneration insufficiency.' The authors cite an older study that does not clearly highlight the extent of cutaneous denervation or the progression of SNAP amplitudes across different disease stages. I would kindly request that the authors revise this sentence, citing a more appropriate and recent study about skin innervation across amyotrophic lateral sclerosis clinical stages.

**Response:** We thank the Reviewer for this constructive feedback on pathological sensory disturbance detected through skin biopsies in ALS. As you pointed out, we have cited other, more recent study and revised the following sentence in the fifth paragraph of the discussion part: “Pathological sensory disturbance detected through skin biopsies has also been reported for ALS, with observations including, a significant loss of intraepidermal nerve fiber (IFNF) and Meissner corpuscle density in ALS compared with healthy controls, and increasing IENF density over time were associated with a poorer prognosis” (line 298-301).
